# New Methods of Treatment in Phthisis

**Published:** 1891-11-07

**Authors:** 


### THE PRACTITIONER'S MIRROR AND RETROSPECT.

[*The Editor will be glad to receive offers of co-operation and contributions from members of the profession. All letters should be addressed to* THE EDITOR, THE LODGE, PORCHESTER SQUARE, LONDON, W.]

### MEDICINE.

### NEW METHODS OF TREATMENT IN PHTHISIS.

#### VI.—THE PICOT METHOD.

In March of this year Dr. Picot, of Bordeaux, first communicated to the Academy of Medicine some results obtained after two months' trial of his method of systematic hypodermic injection of quaiacol and iodoform in cases of phthisis. More recently he brought forward the results of a more extended trial at the recent "Congress of Tuberculosis" at Paris. This method consists in injecting hypodermically a solution containing the active principle of creosote (quaiacol) and iodoform. As the result of many trials, M. Picot succeeded in making a clear solution of these substances in sterilised oil and vaseline. Each centimetre of the solution contains ·01 centigrammes of iodoform and ·05 centigrammes of guaiacol. The injections are made into the supra-spinous fossæ, and never produced any local trouble in the skin and subcutaneous tissues. From one to three centigrammes are injected at a time, and even with the larger quantities, the most that was noticed was some temporary swelling. No general reaction followed the injections, nor any increase in the fever. In some cases, in about half an hour after the injections, a profuse sweating broke out over the body, lasting about an hour and a half, but on its disappearance the patients usually felt better, and the temperature fell half a degree to a degree. In his first communications he reported results in twenty-five cases. Of these, three who were advanced cases and in a very bad condition, died. The fever in these cases had remained uninfluenced, but two of these were relieved in some respects. The post-mortem examinations in these cases revealed some important facts. In the first place there was no sign of congestion having been produced around the old tubercular foci, and there were no signs of fresh crops of tubercles. The cavities contained no casein, nor even fluid matter. A kind of drying process seemed to have taken place in the cavities. In one case there was ulceration of the intestines, and in this case the ulcers, instead of being covered, as is usual, with caseous detritus presented a reddish colour; the edges were even, and seemed drawn towards the centre of the ulcers. The remaining twenty-two cases were all undoubtedly cases of confirmed tuberculosis, which had reached at least the second stage, as demonstrated by stethoscopic signs and the presence of bacilli in the sputum. The results in many of them were most encouraging, and the general health improved wonderfully. In his later communication, M. Picot stated that the patients on which he had employed his method were cases of pulmonary tuberculosis, tubercular pleurisy, and peritoneal tuberculosis.

He had treated forty-two cases of pulmonary phthisis, seven of which were in the last stages. For the latter no further benefit resulted than a diminution in expectoration, and some transient amelioration, which we must remember, all tubercular subjects are liable to show. In other cases, in the third stage, the treatment had produced, after twelve or fifteen injections, an amelioration with diminution of the expectoration, and of the number of bacilli, increased weight and with important modifications of the local physical signs. It is essential that this treatment be continued over a considerable period of time before these improvements are noticeable.

Twenty cases of pulmonary tuberculosis in the second stage were treated, and the results have been variable. In two, in which the lungs were invaded in their whole extent, the treatment had shown itself powerless—at any rate, the lesions had remained *in statu quo*. In two others, in which the mischief was less extensive, he had obtained, on the other hand, an appreciable diminution in the local signs after twelve to fifteen injections. The other patients who had been under the treatment were suffering from phthisis in the first stage. In all these, after fifteen or twenty injections, the expectoration was notably diminished, the appetite restored, the fever had disappeared; but here M. Picot was guarded in claiming a cure, for several patients had already returned with the same signs. "Could these improvements be sustained and the cure remain permanent? Time only will allow of a reply to this question." Further, in these cases M. Picot had the opportunity of treating by these subcutaneous injections three cases in galloping consumption, and in these three he had at least the satisfaction of seeing the rapid course of the disease changed to a chronic form.

In twelve cases of pleurisy, probably tubercular, the effusion disappeared after twelve injections. Only once did the effusion recur, and a second course of injections had resulted in a complete and permanent disappearance of the effusion. At the congress MM. Weil and Diamantberger stated that they had employed injections of quaiacol and iodoform in the treatment of pulmonary tuberculosis. They used a solution of quaiacol in equal parts of almond oil, and they had never observed any local trouble. In fourteen cases in the first stage the benefits had been great, eight were regarded as cured, and six greatly improved. Of eighteen patients in the second stage, three were made worse, two died, and twelve were improved. Of thirteen in the third stage of phthisis the treatment had improved seven very markedly. More recently, at the recent meeting of the British Medical Association, Dr. Robertson, of Peterborough, had made a trial of M. Picot's method in twenty-eight patients, and he arrived at the following conclusions: 1. In empyema one gets satisfactory results after the thorax has been opened, but it is necessary to drain the pleural cavity with care. 2. In pulmonary phthisis with marked elevation of the temperature the injections frequently produce some improvement in lessening the fever and consequently the activity of the morbid processes. 3. Again, when the fever does not diminish to a notable degree, one can induce a diminution in cough and expectoration. 4. The injections do not appear to increase the weight of the patients. 5. The injection of iodoform and quaiacol is not attended with any danger. 6. This treatment does not prevent the development of certain complications, such as meningitis, pleuritis, hæmoptysis, and pneumothorax.

At the same meeting Dr. Shingleton Smith, of Bristol, stated that he considered that the administration of quaiacol and iodoform by the mouth gave equally good results as the injections; one can, in this way, get the patient to take very much larger doses.

The method of M. Picot is but a definite and systematic application of a remedy which is largely employed by physicians of the present day, and we should expect some favourable results. In all the systems which are based on a common-sense application of our pathological and therapeutical knowledge the secret of their measure of success is attention to detail and perseverance in these details for a considerable period.

